# Energy Harvesting from Brines by Reverse Electrodialysis Using Nafion Membranes

**DOI:** 10.3390/membranes10080168

**Published:** 2020-07-28

**Authors:** Ahmet H. Avci, Diego A. Messana, Sergio Santoro, Ramato Ashu Tufa, Efrem Curcio, Gianluca Di Profio, Enrica Fontananova

**Affiliations:** 1 Department of Environmental Engineering, University of Calabria, 87036 Rende (CS), Italy; ahmethalilavci@hotmail.com (A.H.A.); diego.messana.ded@gmail.com (D.A.M.); sergio.santoro@unical.it (S.S.); efrem.curcio@unical.it (E.C.); 2 Institute on Membrane Technology of the National Research Council (ITM-CNR), at University of Calabria, 87036 Rende (CS), Italy; g.diprofio@itm.cnr.it; 3 Department of Energy Conversion and Storage, Technical University of Denmark, Building 310, 2800 Kgs. Lyngby, Denmark; rastu@dtu.dk; 4 SELIGENDA Membrane Technologies SrL, 87036 Rende (CS), Italy

**Keywords:** reverse electrodialysis, Nafion, brine

## Abstract

Ion exchange membranes (IEMs) have consolidated applications in energy conversion and storage systems, like fuel cells and battery separators. Moreover, in the perspective to address the global need for non-carbon-based and renewable energies, salinity-gradient power (SGP) harvesting by reverse electrodialysis (RED) is attracting significant interest in recent years. In particular, brine solutions produced in desalination plants can be used as concentrated streams in a SGP-RED stack, providing a smart solution to the problem of brine disposal. Although Nafion is probably the most prominent commercial cation exchange membrane for electrochemical applications, no study has investigated yet its potential in RED. In this work, Nafion 117 and Nafion 115 membranes were tested for NaCl and NaCl + MgCl_2_ solutions, in order to measure the gross power density extracted under high salinity gradient and to evaluate the effect of Mg^2+^ (the most abundant divalent cation in natural feeds) on the efficiency in energy conversion. Moreover, performance of commercial CMX (Neosepta) and Fuji-CEM 80050 (Fujifilm) cation exchange membranes, already widely applied for RED applications, were used as a benchmark for Nafion membranes. In addition, complementary characterization (i.e., electrochemical impedance and membrane potential test) was carried out on the membranes with the aim to evaluate the predominance of electrochemical properties in different aqueous solutions. In all tests, Nafion 117 exhibited superior performance when 0.5/4.0 M NaCl fed through 500 µm-thick compartments at a linear velocity 1.5 cm·s^−1^. However, the gross power density of 1.38 W·m^−2^ detected in the case of pure NaCl solutions decreased to 1.08 W·m^−2^ in the presence of magnesium chloride. In particular, the presence of magnesium resulted in a drastic effect on the electrochemical properties of Fuji-CEM-80050, while the impact on other membranes investigated was less severe.

## 1. Introduction

The increasing demand for water and energy requires sustainable and environmentally friendly solutions. Therefore, the old-fashioned linear approach (“take, make and dispose”) gives way to the circular economy approach in which any waste is potentially considered as a valuable source for another process. In this regard, reverse electrodialysis (RED) is a promising electromembrane-based process that harvests the Gibbs free energy of mixing of solutions with different salinity [1]. For example, although the brine solution coming from seawater desalination is currently considered as a waste, thanks to its high salinity it can be exploited as a valuable source for RED [2,3,4].

**Figure 1:**
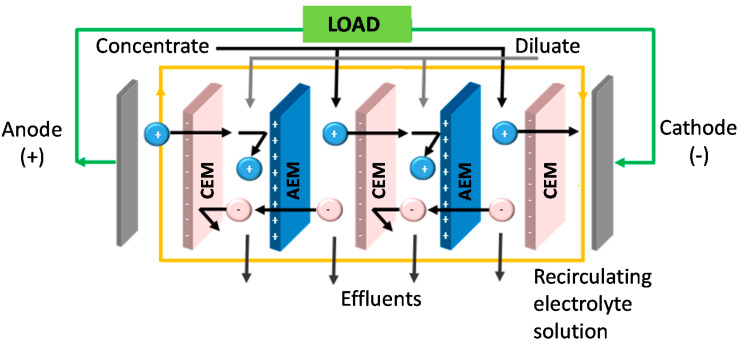
Illustration of the RED process.

A typical RED unit (Figure 1) is similar to an electrodialysis (ED) unit, a well-established and commercialized technology. However, the operating conditions of RED are different. The inputs to ED are a feed solution and the electrical energy, producing separately a concentrate and a dilute. On the other hand, the inputs to RED are a concentrated solution and a dilute solution, mixed together in a controlled manner to produce spontaneously electrical energy [5]. In a RED stack, alternately arranged cation exchange membranes (CEMs) and anion exchange membranes (AEMs) are separated by spacers and piled up in a repetitive organization. When feeding concentrated and diluted solutions throughout the channels created by spacers, a Nernst potential is generated which drives the ions from high electrochemical potential to low electrochemical potential. However, only counter-ions (oppositely charged ions with respect to fixed charge groups of ion exchange membranes) can diffuse through IEM, while co-ions (having the same charge of IEM) are retained. As a result, a steady ion flux occurs between adjacent compartments. Utilization of the appropriate electrolyte solution and electrode couple at the end of compartments allows the transformation of this ion flux into an electric current [6].

IEMs are one of the most important components of a RED stack: in order to maximize generated power, high permselectivity and ion conductivity are essential. Beside these two properties, adequate mechanical strength and low cost are also desired. Moreover, the use of sustainable membrane production protocols for optimizing the green benefits of advanced separation techniques is a key issue of the modern membrane industry [7,8].

So far, numerous researchers readapted IEMs designed for other electrochemical processes (i.e., electrodialysis) to RED process [9]. Due to their high costs, perfluorosulfonic acid polymer electrolyte membranes were not tested before in reverse electrodialysis applications although these membranes are widely used for many applications such as chlor-alkali electrolysis [10,11], water electrolysis [12,13], polymer electrolyte fuel cells [14,15].

**Figure 2:**
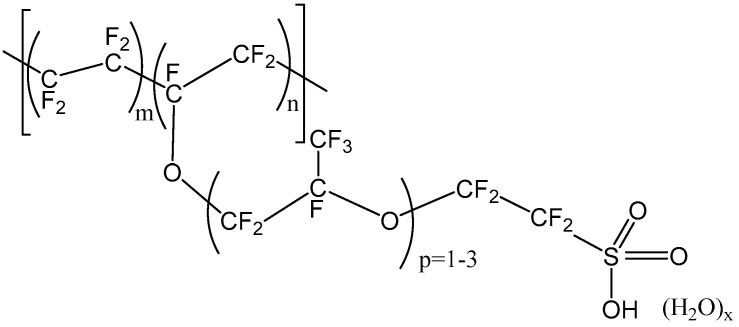
Chemical structure of Nafion.

The chemical structure of Nafion, one of the most commercially relevant perfluorosulfonic acid polymers, is shown in Figure 2 [16]. It is synthesized by perfluorinated vinyl ether comonomer and tetrafluoroethylene copolymerization. The resulting polymer has outstanding long-term chemical and thermal stability. Beside its stability, previous researchers revealed notable permselectivity and conductivity of Nafion membranes in NaCl solutions [17,18,19].

One of the main disadvantages of Nafion membranes is the high cost. For instance, Nafion 117 price was stated between $1400/m^2^ and $2200/m^2^ [20]; Yee et al. (2012) reported a normalized cost of Nafion 117 and Nafion 115 of $3800/m^2^ and $3100/m^2^, respectively [21]. Processing huge volumes of solutions with different salinity requires a large membrane area in RED. Consequently, an elevated capital cost makes the operation economically infeasible. According to Daniilidis et al. (2014), for a 2.7 W·m^−2^ power-producing RED stack having a competitive levelized cost of electricity (LCE) with conventional renewable technologies, the cost of IEM must be around 4 €·m^−2^ [22]. Although the current price of Nafion is far from this estimation, a reduction is projected for large scale production and technological improvements [23].

**Figure 3:**
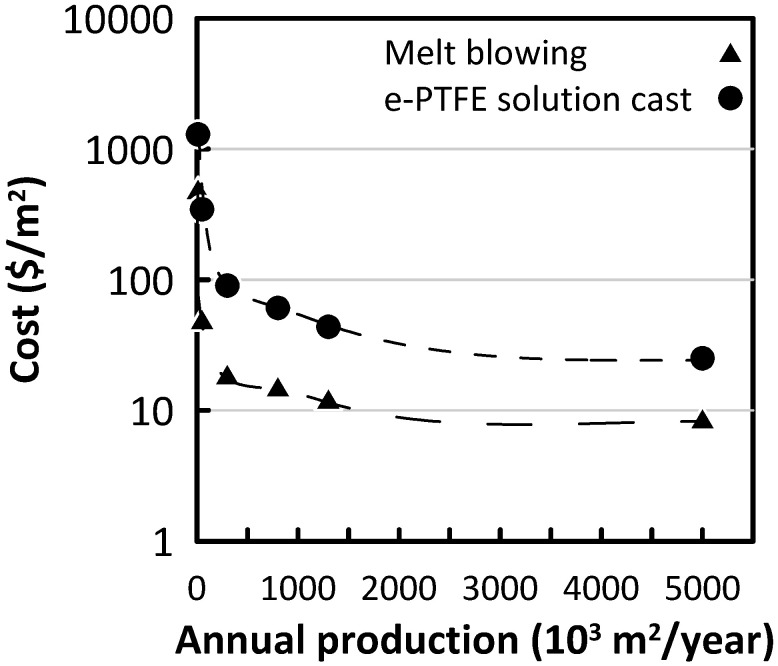
Cost estimation of perfluorinated sulfonic acid polymer electrolyte membrane produced by two different methods as a function of the total yearly production (data from [24]).

Toupin et al. (2016) carried out a study on the cost of Nafion and other perfluorinated sulfonic acid (PFSA) polymer electrolyte membranes to use in fuel cell vehicles; in particular, the cost of membranes was estimated in the case of a different annual production rate for melt blowing and e-PTFE solution cast methods (Figure 3) [24]. Both methods were able to reduce the cost of the membranes by approximately two orders of magnitude while the melt blowing method of production resulted in superior value of 7.7 €·m^−2^ for 5 million m^2^ annual production [24]. Moreover, this value is expected to get lower with increasing technological maturation and production rates.

Other commercial membranes designed for electrochemical processes that are suitable for electrodialysis or RED are expected to be acquired for a lower price compared to Nafion. In this work, CMX Neosepta from Astom Corporation (Tokyo, Japan) and Fuji-CEM-80050 from Fujifilm Manufacturing Europe B.V (Tilburg, the Netherlands) cation exchange membranes were considered as the benchmark for their frequent use in RED application as cation exchange membranes. Unlike Nafion, these membranes are non-perflourinated based membranes. Although the information about preparation methods of these commercial membranes is limited in the literature, it is known that Fujifilm cation exchange membranes have an aliphatic polyamide backbone with sulfonic groups reinforced by uncharged polyolefin support [25,26]. On the other hand, CMX membranes are prepared by the so-called “paste method”: a paste embedded into polyvinyl chloride fabric contains sulfonated styrene monomer, a crosslink agent (i.e., divinylbenzene), polymerization initiator and polyvinyl chloride [26,27].

Different saline solutions have mixed in RED to produce electricity, mainly: fresh water/seawater [5,22,23], seawater/brine [28,29], seawater/groundwater [30], brackish water/brine [31,32,33]. Among them, mixing seawater and brine (the waste from the desalination process) solutions have operational, economic, and environmental advantages. Due to low electrolyte concentration in fresh water and brackish water, the conductivity of these solutions limits the efficient transportation of ions within a compartment. Conversely, utilizing seawater in the low concentration compartments reduces the total stack resistance and increases the generated power.

Studies over the past decade have provided important information on seawater/brine mixing by RED. Daniilidis et al. (2014) investigated the performance of Neosepta CMS and ACS in RED for a wide range of NaCl solutions: for 0.5 M/5.0 M NaCl mixing, 1.5 W·m^−2^ gross power density was detected. It is noteworthy that permselectivity was about 20% lower when compared to 0.1/0.5 M NaCl feed, while a fourfold decrease in stack resistance was observed compared to 0.01 M/0.5 M NaCl feed [22]. In a study on an integrated membrane distillation–reverse electrodialysis system, for a RED stack equipped with Fuji-CEM 80050 and AEM 80045 and operated with 0.5 M/4.0 M NaCl feed, Tufa et al. (2015) measured an open circuit voltage (OCV), a stack resistance (R_stack_) and a gross power density (P_d_) of 1.25 V, 7 Ω·cm^2^ and 0.9 W·m^−2^, respectively [4].

With a share of ~10%, magnesium is the second most abundant cation in seawater [34]. Despite its importance, the effect of Mg^2+^ on RED performance at high salinity is still poorly investigated [35]. In one of these studies, Avci et al. (2016) observed a 20% and a 60% reduction of OCV and power density, respectively, when 10% molal MgCl_2_ was present in feed solutions. It was also noted that the reason for significant power loss can be attributed to tripled resistance of Fuji-CEM-80050, while no notable change was observed for Fuji-AEM-80045 [36]. Similarly, Fontananova et al. (2017) compared the electrochemical properties of the abovementioned membranes in analogous operative conditions: 40% loss in permselectivity and 3.5 times higher resistance was observed for CEM, while AEM permselectivity decreased only by 16% with resistance remaining almost stable. Consequently, gross power density reduced from 0.96 to 0.67 W·m^−2^ [37].

The main purpose of this study is to characterize the electrochemical properties of Nafion membranes for RED operations carried out at high salinity gradients and compare them with commercially available non-perflourinated membranes frequently utilized in RED. For this reason, Nafion 117, Nafion 115, CMX and Fuji-CEM-80050 were characterized by electrochemical impedance spectroscopy (EIS) and potential cell for NaCl and NaCl + MgCl_2_ solutions with ionic strengths mimicking seawater (0.5 mol·kg^−1^) and hypersaline brine (4.3 mol·kg^−1^).

To the best of our knowledge, this is the first work in which Nafion membranes were tested in a RED stack, although numerous works were carried out in different fields such as fuel cells and chlor-alkali processes.

## 2. Materials and Methods

### 2.1. Feed and Electrolyte Solutions

Three different solutions are required to operate reverse electrodialysis: a high concentration compartment (HCC) solution, a low concentration compartment (LCC) solution, and an electrolyte compartment solution. Solutions for RED experiments and electrochemical characterization were prepared by dissolving appropriate amounts of NaCl, MgCl_2_·6H_2_O, K_4_[Fe(CN)_6_]·3H_2_O and K_3_[Fe(CN)_6_] (supplied by Sigma-Aldrich, Milan, Italy) in deionized water (0.055 µS·cm^−1^, produced by PURELAB, Elga LabWaters, High Wycombe, United Kingdom). The compositions of the solutions are reported in Table 1.

The ionic strength I (mol/kg of water) of a solution is calculated as:

(1) $$I=\frac{1}{2}\sum m_{i}z_{i}^{2}$$

where *m_i_* and *z_i_* are the molality (i.e., moles solute per kg of solvent) and the charge of the *i*-th ion, respectively.

A 3M KCl (Sigma-Aldrich, Milan, Italy) was prepared to fill Haber-Luggin capillaries in EIS measurements.

### 2.2. Membranes

Nafion 117 and Nafion 115 were purchased from Quintech (Göppingen, Germany). Fuji CEM 80050 (hereinafter referred to as “Fuji-CEM”) was kindly supplied by FujiFilm Manufacturing Europe B.V. ((Tilburg, The Netherlands). Neosepta CMX and AMX were kindly supplied by Eurodia (Pertuis, France).

All dry membrane samples were initially activated in 0.5 M NaCl solution. Additionally, they were conditioned in the specific test solution before use. For example, prior to the electrochemical impedance characterization in 4 M NaCl, membrane samples were immersed in this solution for at least 24 h and the solution was changed at least 3 times during this period.

### 2.3. Membrane Permselectivity

**Figure 4:**
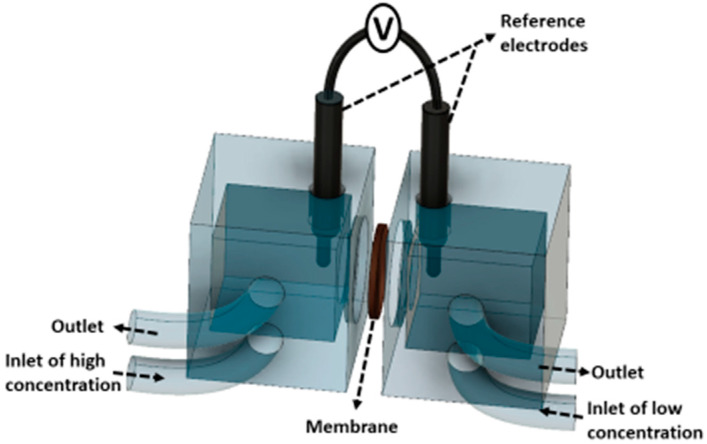
Scheme of the two compartments/two electrodes cell used for membrane potential measurements.

The membrane potential was measured by using two Ag/AgCl reference electrodes (Gamry Instruments, Warminster, PA, US) as in Figure 4. DC voltage drop across the membrane was recorded by a digital multimeter in the range of 0 to 600 mV (Fluke 117, Fluke Corporation, Everett, WA, US). Membrane potential of CEMs were characterized in two different solution pairs: 0.5/4.0 M NaCl and 0.34 M NaCl + 0.054 M MgCl_2_/2.72 M NaCl + 0.43 M MgCl_2_. Test solutions were kept at 25 ± 3 °C and fed to the cell at a flow rate of 1.5 cm·s^−1^.

After obtaining the membrane potential experimentally (Δ*V_exp_*), permselectivity (α) was calculated by taking the ratio to theoretical membrane potential (Δ*V_theo_*):

(2) $$\alpha =\frac{∆V_{exp}}{∆V_{theo}}$$

The theoretical membrane potential was calculated by the Nernst equation [38]:

(3) $$∆V_{theo}=\sum \frac{RT}{z_{i}F}ln\frac{\gamma_{i}^{c}c_{i}^{c}}{\gamma_{i}^{d}c_{i}^{d}}$$

where *R* is the universal gas constant (8.3144 J·K^−1^mol^−1^), *T* is the temperature (K), *z* is the valence number (−), *F* is the Faraday constant (96485 C·mol^−1^), *γ* is the activity coefficient and *c* is the molality. Subscript *i* stands for the component type, while superscripts *c* and *d* refer to the concentrated solution and the diluted solution, respectively. The activity coefficients were calculated by the interpolation from experimental values [39].

### 2.4. Electrochemical Impedance Spectroscopy (EIS)

**Figure 5:**
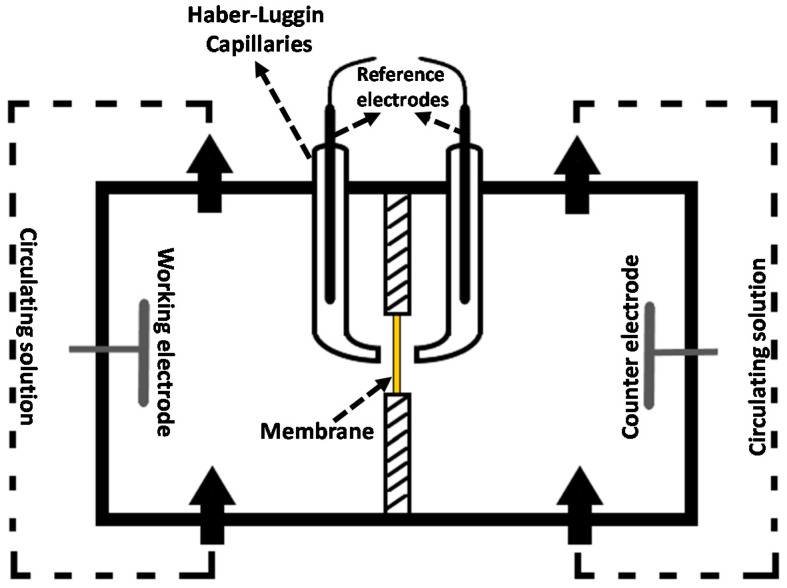
Scheme of the two compartments/four electrodes electrochemical impedance cell.

In order to characterize ohmic and nonohmic resistance of a membrane-solution system, EIS experiments were carried out with a potentiostat/galvanostat combined with a frequency response analyzer (PGSTAT302N, Metrohm Autolab B.V., Utrecht, The Netherlands). As it is shown in Figure 5, a specifically designed four-electrode configuration was used in the impedance cell with 3.14 cm^2^ active membrane area [40]. An alternating current in the frequency range 1000–0.01 Hz, with a signal amplitude of 10 mV, was applied between working and counter electrodes (made of Ag), while the response (voltage drop) was measured by the reference electrodes immersed in the Haber−Luggin capillaries containing 3M KCl solution.

**Figure 6:**
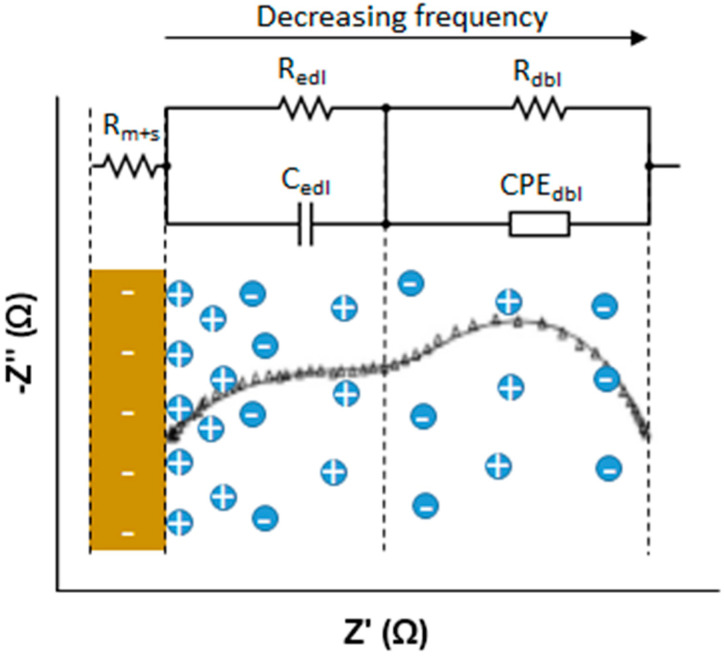
Equivalent circuit of a membrane-solution system impedance on the Nyquist diagram.

The response of the membrane solution system was plotted into a Nyquist diagram and fitted to the equivalent circuit shown in Figure 6, generated by the software Nova 1.9.16 (from Metrohm Autolab B.V., Utrecht, The Netherlands). The membrane-solution resistance is an ohmic resistance obtained from the intersection point of the curve and −Z” = 0 at high frequency. In order to calculate stand-alone membrane resistance, repetition of the experiment under the same conditions without the membrane was required; by subtracting solution resistance from the membrane-solution resistance, membrane resistance could be determined. On the other hand, electrical double layer resistance (EDL) and the diffusion boundary layer (DBL) cannot be modelled by only resistance due to their electrochemical nature. As illustrated in Figure 6, EDL consists of a resistance and a capacitance in parallel while DBL consists of a resistance and a constant phase element in parallel. Both of them appeared as a semi-circle in the Nyquist plot at different frequency ranges: EDL at medium frequencies whereas DBL at low frequencies.

The EIS experiments were carried out at 25 °C and 1.5 cm·s^−1^ by circulating the LCC and HCC solutions individually.

### 2.5. Water Uptake, Ion Exchange Capacity and Fixed Charge Density

Membrane water uptake (WU) was calculated by weighing the membrane swelled in 0.5 M NaCl solution (*w_swelled_*) and dry membrane (*w_dry_*);

(4) $$WU\%=\frac{w_{swelled}-w_{dry}}{w_{dry}}\cdot 100$$

Ion exchange capacity of CEMs were calculated as reported previously [37]. In order to saturate negative fixed charge groups of CEMs, samples were kept in excess 1 M HCl solution overnight; then to remove all uncoupled H^+^ present in the surface water, the samples were washed with demi-water. Following this, H^+^ ions were exchanged with Na^+^ ions by immersing the samples into 40 mL of 2 M NaCl. Finally, the immersed solutions were collected into a beaker and titrated with 0.01 M NaOH. The pH values were monitored with a pH meter (WTW Inolab Terminal Level 3, Weilheim, Germany). The *IEC* (meq∙g dry membrane^−1^) was calculated by using the following equation:

(5) $$IEC=\frac{V_{NaOH}\cdot M_{NaOH}}{m_{dry}}$$

in which *V_NaOH_* is the volume of NaOH titrant (l), *M_NaOH_* is the molarity of NaOH titrant (mol∙L^−1^) and m_dry_ is the dry weight of the sample (g) after washing with water and leaving in an oven at 70 °C overnight.

The fixed charge density (*C_fix_*) was calculated by using water uptake, *IEC* values and water density at 25 °C (*d_w_*):

(6) $$C_{fix}=\frac{IEC\cdot d_{w}}{wu\%}\cdot 100$$

### 2.6. Reverse Electrodialysis

The lab-scale electrodialysis cell PCCell 200, provided by PCCell GmbH (Heusweiler, Germany), was used in reverse electrodialysis mode to characterize electrochemical performance of the stack equipped with the aforementioned CEMs paired with AMX Neosepta. CEMs were cut into 26.2 × 12.5 cm^2^ pieces to fit 500 μm thick spacers for 207 cm^2^ total active area. The electrode compartments included anode and cathode made of inert Pt/Ir-coated titanium mesh. The electrode compartments were separated from the central compartments by CMX membranes. Between the central membrane and these CMX membranes, AMX membranes were utilized as anion exchange membranes.

The performance of the RED unit was investigated at 25 °C and the linear flow velocity of the concentrated and diluted compartments was 1.5 cm·s^−1^. Flowrate of electrolyte solution was fixed to 30 L∙h^−1^. Solutions were fed by Masterflex L/S digital peristaltic pumps (Cole-Palmer, Vernon Hills, IL, US) and conditioned to the desired temperature by a refrigerated/heated circulating bath (PolyScience, Niles, IL, US) before entering the stack. Two different salinity gradients were tested: 0.5 M/4.0 M NaCl and 0.34 M NaCl + 0.054 M MgCl_2_/2.7 M NaCl + 0.43 M MgCl_2_ Table 1.

The current (I) versus voltage (V) curve, that is linear coherently with Ohmic law, was plotted by applying DC current by Methrom Autolab in the range of 0–32 A/m^2^. Open circuit voltage (OCV) was obtained from both fitted data (at I = 0 A) and experimental measurements, while stack resistance (*R_stack_*) was calculated from the slope of I-V curve. Then, gross power density (*P_d_*, W·m^2^) and current density (A_d_, I·m^2^) were determined and fitted as a parabola.

In line with the ohmic behavior of RED, gross power density *P_d,max_* is proportional to the OCV^2^ and reversely proportional to *R_stack_*:

(7) $$P_{d,max}=\frac{OCV^{2}}{4N\cdot R_{stack}}$$

The maximum power density (*P_d,max_*) was calculated from the maximum of parabola.

## 3. Results

Ion exchange membranes have a great importance for energy conversion from salinity gradients by reverse electrodialysis [41]: the power potential of a RED unit, estimated from OCV and *R_stack_*, is strictly related to permselectivity and electrical membrane resistance. In turn, these properties are interrelated to other characteristics, i.e., thickness, ion exchange capacity (IEC), water uptake (WU), and fixed charge density (*C_fix_*) (Table 2). It is difficult to have a straightforward comment on the effect of a single IEM property due to strong interconnections and counteractions among all of them. For example, high IEC is a way to reduce the resistance. However, since water uptake increases with increasing IEC, the concentration of fixed charged groups attached to the polymeric matrix decreases, thus reducing permselectivity. A significant increase of IEC also results in swollen and mechanically weak membranes.

The thickness of CEMs used in this study ranges between 114–201 µm, which is typical for CEMs used previously for RED [42]. Even though the thickness and the ionic resistance are proportional, thinner membrane does not necessarily perform better. Tedesco et al. (2018) carried out experiments with FAS and FKS Fumasep (FUMATECH BWT GmbH, Bietigheim-Bissingen, Germany) membranes with varying thickness between 14–90 µm, and concluded having thinner membranes was not beneficial for maximum power density [43].

Referring to Table 2, although Nafion 115 and 117 membranes exhibited relatively lower IEC, low water uptake made *C_fix_* superior compared to the investigated benchmark membranes. Conversely, high WU and moderate IEC of the Fuji-CEM membrane resulted in the lowest *C_fix_.*

### 3.1. Electrochemical Properties of CEMs

#### 3.1.1. Permselectivity

For ion exchange membranes, the permselectivity is an indication of the ability to selectively transport counter-ions over co-ions. To be able to control the mixing of ions in a preferred direction during a RED process, a permselectivity higher than 0.95 is desired [44]. Most of the reported commercial CEMs have acceptable permselectivity in this regard. However, generally, permselectivity characterization is carried out in 0.1/0.5 M NaCl or KCl, which is not representative for high concentration or complex solutions with multivalent ions used in real cases. Therefore, in this study, permselectivity of Nafion 115, Nafion 117, CMX and Fuji-CEM were characterized for concentrated (ionic strength > 0.5 mol·kg^−1^) and multicomponent solutions (Table 1).

**Figure 7:**
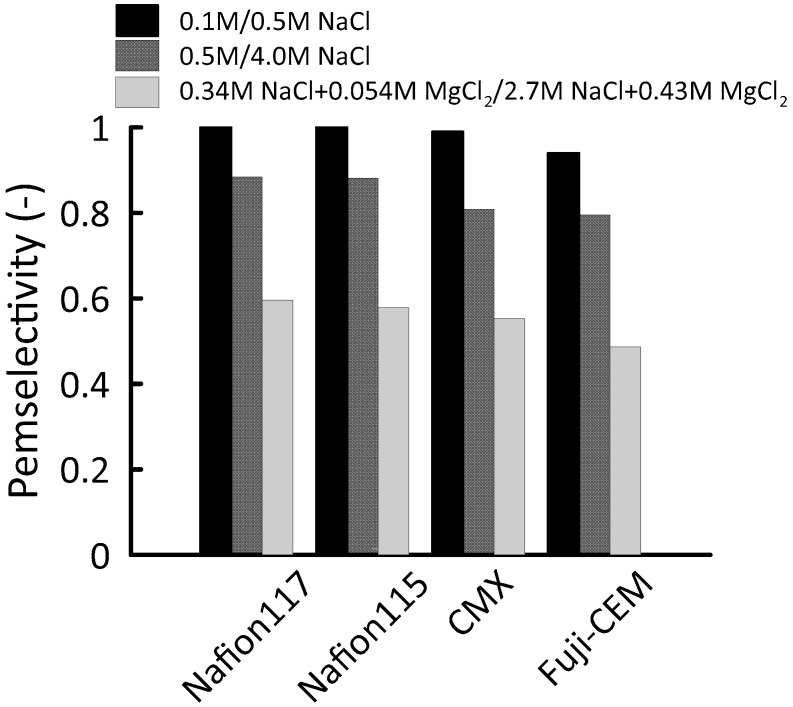
Permselectivity of Nafion 117, Nafion 115, Neosepta CMX, Fuji-CEM-80050 in −0.1/0.5 M NaCl, 0.5/4.0 M NaCl and 0.34 M NaCl + 0.054 M MgCl_2_/2.7 M NaCl + 0.43 M MgCl_2_ at 25 °C.

Figure 7 compares the permselectivity (α) of the membranes at 25 °C. In standard 0.1/0.5 M NaCl test solution pairs, all membranes performed satisfactorily enough for a RED application; Nafion membranes characterized as ideal (1.0) while CMX resulted in almost ideal (0.99) and Fuji-CEM had sufficient permselectivity (0.94). Having *C_fix_* around 8 mol·L^−1^, Nafion membranes exhibited high co-ion exclusion with pure NaCl solutions, even when one side of the membrane was in touch with 4.0 M NaCl (α = 0.88) whereas the permselectivity of CMX and Fuji-CEM membranes was 8% and 10% lower, respectively. This deviation from unity is in accordance with the previous literature data [28,37]. The co-ion equilibrium in an ion exchange membrane for ideal monovalent electrolyte can be expressed as the following equation:

(8) $$C_{co}^{m}=\frac{C_{co}^{2}}{C_{fix}^{m}}$$

where *C* is the concentration, *m* stands for membrane, subscript “*co*” and “*fix*” are co-ion and fixed charge, respectively. From Equation (8), it can be deduced that a low fixed charge concentration leads to a lack of co-ion exclusion when the membrane is exposed to a high concentration of electrolyte.

A more detrimental effect on permselectivity was observed with the introduction of MgCl_2_ to the electrolyte solution. The losses of permselectivity were recorded between 32–38%; the lowest permselectivity (α = 0.49) was measured for Fuji-CEM. In general, this drastic reduction can be explained by investigating the binding affinity of counter-ions [45]. With an increasing binding affinity of a counter ion/fixed charge group, the possibility of condensation of the counter-ion increases. Therefore, counter-ion concentration in the ionic state decreases as long as the neutralization of the fixed charge groups occurs. Consequently, co-ion transport across the membrane increases. According to Luo et al. (2018), the divalent cations affinity to sulfonic groups is significantly higher than the Na^+^ [46].

#### 3.1.2. Electrochemical Impedance

**Figure 8:**
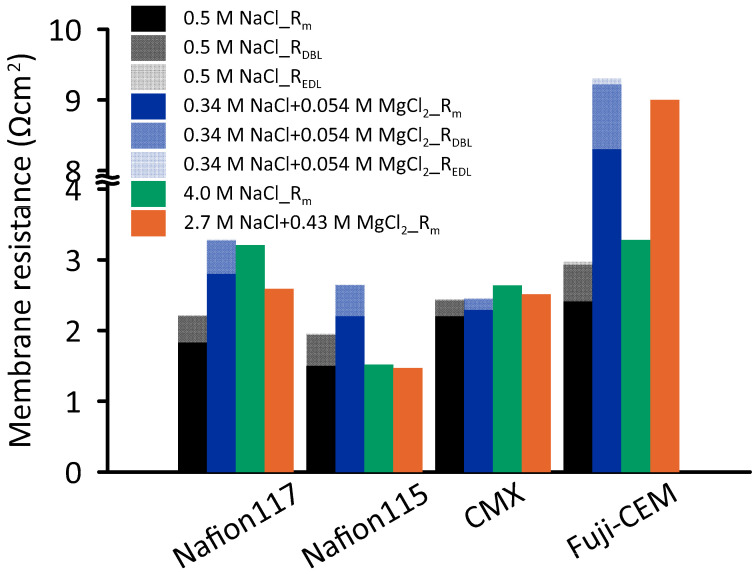
Ionic resistance of Nafion 117, Nafion 115, CMX, Fuji-CEM-80050 in 0.5 M NaCl, 4.0 M NaCl, 0.34 M NaCl + 0.054 M MgCl_2_ and 2.7 M NaCl + 0.43 M MgCl_2_ at 25 °C.

The total RED stack resistance consists of CEMs, AEMs, HCC, LCC and electrolyte compartment resistances [47]. When RED is operated with seawater and river water, LCC resistance dominates the total resistance [48]. However when using high concentrated feed solutions, the contribution of ionic membrane resistance becomes critical in understanding the RED performance [49]. Electrochemical impedance test is a powerful technique to quantify not only the membrane resistance, but also the boundary layer resistance at the membrane solution interface. Figure 8 illustrates the resistance of four CEMs against four different concentrations. For 0.5 M NaCl, the lowest resistance (1.50 Ω·cm^2^) was measured for Nafion 115, while 15% higher resistance was obtained for Nafion 117. The resistance of CMX and Fuji-CEM were 2.20 and 2.41 Ω·cm^2^, respectively. These findings are comparable with those of Fontananova et al. [40] and Galama et al. (2016) [49] who measured Fuji-CEM and CMX resistance as 2.97 and 2.58 Ω·cm^2^, respectively. Although thickness and water uptake values are in favor of Fuji-CEM compared to the others, the low charge density determines relatively high resistance.

Interestingly, at 4.0 M NaCl, the resistance of Nafion 115 remained constant compared to the resistance at 0.5 M NaCl, whereas other CEM resistances increased 20–75%. It seems possible that these results are due to the decreasing water content in CEMs [37]: with decreasing water uptake, the interstitial channels through the membrane cross section get narrower, so the ionic transfer is impeded.

Due to the significant amount of magnesium in seawater, characterizing the resistance of CEMs in presence of Mg^2+^ ions by EIS provides deeper understanding on RED performance. EIS tests were carried out in NaCl + MgCl_2_ solutions having ionic strength equal to 0.5 M and 4.0 M pure NaCl solutions (Table 1). As shown in Figure 8, the presence of magnesium in the test solution significantly affected the Fuji-CEM conductivity: the resistance increased by 3.4 and 2.7 times when the ionic strength was 0.51 mol·kg^−1^ and 4.3 mol·kg^−1^, respectively. The ionic conductivity of Nafion membranes was halved while no significant effect was observed on CMX at ionic strength of 0.51 mol·kg^−1^. The observed increase in resistance could be attributed to the binding affinity of Mg^2+^, as in the case of permselectivity. As discussed by Cassady et al. (2016), counter-ions in the membrane lattice can exist as a solvated pair or as a condensed salt, having prevalence of the latter form when binding affinity is higher. Consequently, a fixed charge in a condensed salt is electrically neutralized, does not facilitate the counter ion transport anymore, and IEM conductivity reduces [45].

EIS results revealed that, at high salinity, the *R_EDL_* and *R_DBL_* were insignificant compared to *R_m_*. Conversely, for 0.5 M NaCl, interfacial (nonohmic) resistances contributed by 10–23% to the total resistance, while in the presence of magnesium at equivalent ionic strength of 0.51 mol·kg^−1^, the contribution varied from 7% to 17%.

At lower concentrations, the nonohmic resistance is more significant and its contribution can reach 50% [37,50] For example, in 0.1M NaCl solution, the total resistance of Fuji-CEM 80050 resulted in around 4.6 Ω·cm^2^ in which approximately 2.4 Ω·cm^2^ was contributed by the diffusion boundary layer and electrical double layer resistances [50].

With respect to the extent of nonohmic resistances, CMX was found less prone under the investigated conditions. In general, *R_DBL_* was the dominant nonohmic resistance with more than 90% for all CEMs.

In order to diminish the effect of the diffusion boundary layer, several studies were focused on enhancing the fluid mixing in feed compartments. In one of these studies, Guler et al. (2014) prepared microstructured membranes in order to eliminate the usage of spacers: increasing flow rate from 2 to 40 mL·min^−1^ resulted in minimal nonohmic resistance [51]. However, it should be noted that increasing the flow rate leads to a reduction of net power density. Vermaas et al. (2011) investigated the net power density of a RED stack equipped with FKS and FAS (Fumatech) membranes by using different spacer thickness (60, 100, 200 and 485 µm). Each spacer resulted in its maximum at a different Reynold number; for example when 100 µm-thick spacers were used, maximum net power density was measured for *R_e_* = 0.5 while maximum gross power density was obtained for *R_e_*~2.0 [48].

#### 3.1.3. Reverse Electrodialysis Performance

**Figure 9:**
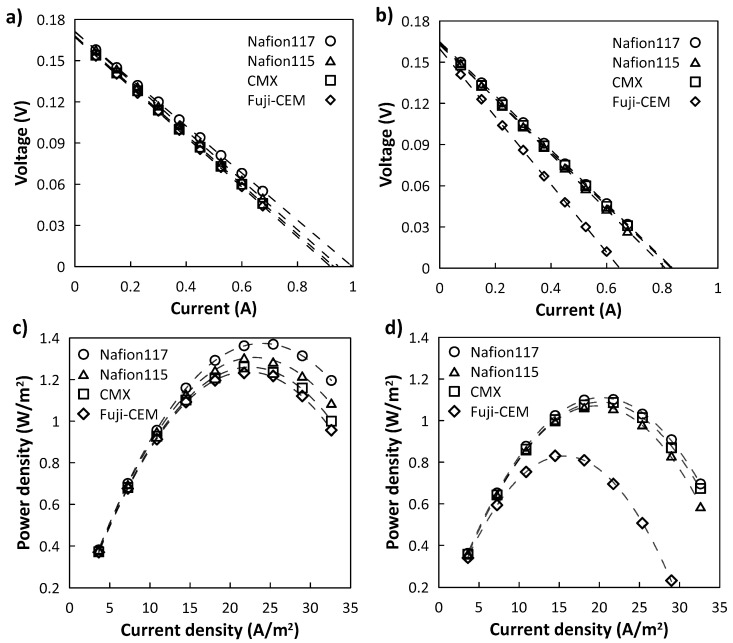
RED performance of the four commercial CEMs investigated: (**a**,**b**) voltage versus current; (**c**,**d**) gross power density versus current density. For (**a**,**c**) test in 0.5/4.0 M NaCl; for (**b**,**d**) test with 0.34 M NaCl + 0.054 M MgCl_2_/2.7 M NaCl + 0.43 M MgCl_2._

Figure 9 illustrates the comparison from electrochemical tests of four different commercial CEMs utilized in the RED stack for solutions at two different compositions as detailed in Table 1.

From the current-voltage curve (Figure 9a,b), the OCV was in the range of 0.167–0.171 V when using pure NaCl solution; the addition of MgCl_2_ resulted in a slight narrowing of this range, i.e., 0.160–0.164 V. In both cases, the decreasing order of OCV was Nafion 117 > Nafion 115 > CMX > Fuji-CEM.

Using a single membrane pair to test RED is the main reason for such a slight variation of OCV; a higher number of membrane pairs enhances the voltage drop across the stack and makes this difference explicit. Even so, the OCV of the RED stack was in line with the permselectivity of CEMs.

The total stack resistance consists of individual resistances that constitute the RED system:

(9) $$R_{stack}=N\left(R_{CEM}+R_{AEM}+R_{HCC}+R_{LCC}\right)+R_{EL}$$

where *N* is the number of the membrane pair and subscript EL stands for electrolyte. In most cases, when large numbers of membrane pairs are used, *R_EL_* is neglected; in this study, being RED operated for a single cell (*N* = 1), this effect has to be considered. Therefore, *R_stack_* lined up very close for all CEMs when feeding 0.5 M/4.0 M NaCl to RED: the lowest and the highest measured *R_stack_* were 0.1709 and 0.1818 Ω for Nafion 117 and Fuji-CEM, respectively. On the other hand, when feeding 0.34 M NaCl + 0.054 M MgCl_2_/2.7 M NaCl + 0.43 M MgCl_2_ solutions, a 35% increase in the *R_stack_* of Fuji-CEM was detected; this increase was limited to 8–15% for the other investigated membranes. This finding corroborates the results obtained in EIS characterization.

Figure 9c,d show that, whether magnesium was present or not, the best and worst performing membranes in terms of maximum power density were Nafion 117 and Fuji-CEM, respectively. When the RED stack was equipped with Nafion 117, gross power density of 1.38 and 1.08 W·m^−2^ were measured for NaCl and NaCl + MgCl_2_ solutions; correspondingly, 1.24 and 0.824 W·m^2^ were obtained with Fuji-CEM. The significant *P_d_* reduction is attributed to loss in both permselectivity and conductivity of the membrane. Moreover, the main reason why Fuji-CEM differed from the others can be explained by analyzing Equation (8). The low fixed charge density of Fuji-CEM (3.2 mol·L^−1^) makes it vulnerable to high salinities whereas having high fixed charge density helps maintain the exclusion capacity, as in the example of N117 (8.0 mol·L^−1^).

These results are coherent with our previous studies on the effect of Mg^2+^. Avci et al. (2016) performed experiments using similar salinity gradients in a RED stack equipped with 25 cell pairs of Fuji AEM 80045 and Fuji CEM 80050: with a 20% reduction in OCV and 60% increase in *R_stack_*, gross power density was more than halved. Specific investigations revealed that the power loss was substantially due to the critical effect of Mg^2+^ on the performance of Fuji CEM [36]. Similarly, Fontananova et al. (2017) reported a 30% decrease in *P_d,max_* for the same concentration of feed solution used in this study when the stack was equipped with 25 pairs of AEM 80045 and Fuji CEM 80050 [37].

Nafion and CMX membranes exhibited very similar performance.

For a better comparison of electrochemical performance of the commercial CEMs tested, permselectivity, membrane resistance from EIS tests, stack resistance, open circuit voltage from RED experiments and resulting maximum gross power density are reported in Table 3.

Regarding the permselectivity, CEMs suffered from the presence of magnesium showing a very clear and sharp decrease. Consequently, OCV values declined since it is proportional to average permselectivity of AEM and CEM.

An increase in *R_stack_* by 11–18% was observed for the stacks equipped with Nafion 115, Nafion 117 and CMX, whereas *R_stack_* increased by about 40% when using Fuji-CEM.

An increase of both *R_HCC_* and *R_LCC_* contributed to the general enhancement of *R_stack_*. In fact, the conductivity of LCC solution reduced from 47.9 to 40.2 mS·cm^−1^ when the feed solution was changed from 0.5 M NaCl to 0.34 M NaCl + 0.054 M MgCl_2_, respectively. Likewise, HCC conductivity reduced from 270.7 to 200.3 mS·cm^−1^ when the feed solution was changed from 4.0 M NaCl to 2.7 M NaCl + 0.43 M MgCl_2_, respectively.

Furthermore, for Fuji-CEM, an additional relevant contribution was associated to the drastic increase in membrane resistance (Figure 8), rising from 2.4 to 8.3 Ω·cm^2^ when changing LCC solution from 0.5 M NaCl to 0.34 M NaCl + 0.054 M MgCl_2_ and, analogously, rising from 3.3 to 9.0 Ω·cm^2^ when changing LCC solution from 4.0 M NaCl to 2.7 M NaCl + 0.43 M MgCl_2_.

Therefore, considering the Nernst Equation (3), stack resistance Equation (9) and maximum gross power density Equation (7), it can be concluded that the experimental single-cell RED parameters were in line with CEMs characterization.

Although this study aims to compare the potential of Nafion-based membranes with typically used CEMs for RED, further optimization of stack components can boost the generated energy. For example, reducing spacer thickness allowed enhanced gross power density to be obtained by decreasing stack resistance [48]. Similarly, a favorable response with less impact to spacer thickness reduction would be expected in this study as well, since feed salinity is high enough to provide required conductivity.

## 4. Conclusions

The present study was designed to elucidate the possible utilization of perfluorosulfonic acid based Nafion in reverse electrodialysis under a high salinity gradient. Additionally, by investigating the effect of magnesium ions, we extended this study from paradigmatic NaCl solutions to multicomponent NaCl + MgCl_2_ solutions in view of a more realistic approach to RED operations in natural environments. In this regard, single cell RED experiments were carried out by using Nafion 117, Nafion 115, CMX and Fuji-CEM-80050 as cation exchange membranes. When operating with 0.5 M/4.0 M NaCl solutions, Nafion membranes resulted in the highest *P_d,max_* thanks to their outstanding permselectivity compared to other CEMs. In the presence of magnesium, 17 and 20% *P_d,max_* reductions were recorded for Nafion 115 and Nafion 117, respectively; both membranes maintained their low resistance, while a significant loss in permselectivity was measured. Even so, Nafion membranes outperformed other commercial membranes such as CMX and Fuji-CEM-80050.

Although Nafion membranes exhibited better performance than CMX and Fuji-CEM-80050, their use is limited by high cost, and a significant reduction of membrane price is required for affordable RED applications.

## Figures and Tables

**Table 1:** Concentration and ionic strength of feed and electrolyte solutions used in RED.

Compartment	Composition	Ionic Strength (mol·kg^−1^)
**LCC**	0.5 M NaCl	0.51
**HCC**	4.0 M NaCl	4.3
**LCC**	0.34 M NaCl + 0.054 M MgCl_2_	0.51
**HCC**	2.7 M NaCl + 0.43 M MgCl_2_	4.3
**Electrolyte**	0.3 M K_4_[Fe(CN)_6_] + 0.3 M K_3_[Fe(CN)_6_] + 2.5 M NaCl	7.3

**Table 2:** Relevant physical and electrochemical properties of membranes at 25 °C.

Membrane	Thickness (µm)	IEC (meq·g^−1^)	Water Uptake (%)	Charge Density (mol·L^−1^)
**Nafion 115**	139 ± 8	0.90 *	11.2 ± 0.02	8.0
**Nafion 117**	201 ± 4	0.90 *	11.7 ± 0.01	7.7
**Fuji-CEM-80050**	114 ± 2	1.1 ± 0.1	34.0 ± 0.00	3.2
**CMX**	166 ± 1	1.61 ± 0.03	25.5 ± 0.1	6.3 ± 0.23

* From the manufacturer.

**Table 3:** Electrochemical characterization data from single-cell RED experiments.

	EIS Tests						Single-Cell RED Tests					
	Permselec. (-)		Membrane Resist. (Ω·cm^2^)				OCV (V)		*R_stack_* (Ω)		*P_d,max_* (W·m^−2^)	
CEM	A/B	C/D	A *	B *	C *	D *	A/B	C/D	A/B	C/D	A/B	C/D
Nafion 115	0.88	0.60	1.5	1.5	2.2	1.5	0.171	0.164	0.18	0.20	1.30	1.08
Nafion 117	0.88	0.58	1.8	3.2	2.8	2.6	0.171	0.165	0.17	0.20	1.38	1.11
CMX	0.81	0.55	2.2	2.6	2.3	2.5	0.168	0.163	0.18	0.20	1.26	1.09
Fuji-CEM	0.79	0.49	2.4	3.3	8.3	9.0	0.167	0.160	0.18	0.25	1.24	0.82

* A: 0.5 M NaCl; B: 4.0 M NaCl; C: 0.34 M NaCl + 0.054 M MgCl_2_ and D: 2.7 M NaCl + 0.43 M MgCl_2_.
